# Book Review: Recent Advances in Yeast Metabolic Engineering

**DOI:** 10.3389/fbioe.2017.00071

**Published:** 2017-11-13

**Authors:** Nisarg Gohil, Happy Panchasara, Shreya Patel, Robert Ramírez-García, Vijai Singh

**Affiliations:** ^1^Synthetic Biology Laboratory, Department of Microbiology, School of Biological Sciences and Biotechnology, Institute of Advanced Research, Gandhinagar, India

**Keywords:** yeast metabolic engineering, promoters, directed evolution, genome-scale metabolic models, metabolomics

Recent advances in the yeast molecular biology toolbox and the access to the complete genome sequencing data of yeast *Saccharomyces cerevisiae* has opened up new avenues for greater understanding of the yeast physiology, metabolism and also has driven the acceleration of its metabolic engineering. Metabolic engineering has the potential to produce high amounts of desired product from renewable resources through the implementation of heterologous biosynthetic pathways or the modification of endogenous pathway. Currently, the easy manipulation of yeast is assisted by the availability of a genetic engineering toolbox, libraries of synthetic promoters, ribosome binding sites, degradation tags, transcription terminators, plasmids, riboregulators, and riboswitches that have been designed and well characterized in yeast (Curran et al., 2014; Tsai et al., 2015).

The molecular toolbox helps us to make yeast a microbial cell factory for the production of a wide range of products. The yeast has been engineered to produce biopharmaceuticals (insulin, enzymes, vaccines), polymers, monomers (lactic, succinic, *cis*,*cis*-muconic acids), fuels, chemicals, nutraceutical ingredients, probiotics, and fuel cells (Borodina and Nielsen, 2014; Kavšček et al., 2015). These yeast cell factories can be engineered using metabolic engineering through the use of systems and synthetic biology principles and by following iterative step wise operation starting from an *in silico* design, followed by construction and the analysis of final variants with the ability to produce desired molecules (Kim et al., 2012). This iterative cycle, which includes the design, build, and test cycle, allows for the development of specialized strains for optimizing bioproduction. In the past decade, yeast research has been boosted and its perspectives have been further augmented by the creation of a synthetic yeast cell of *S. cerevisiae* with a highly modified genome that has reduced (8%) by size (Richardson et al., 2017).

Currently, the type II CRISPR-Cas9 is a key technology for targeted genome editing and regulation in wide range of organisms and cell types (Jinek et al., 2012, 2013; Cong et al., 2013; DiCarlo et al., 2013; Mali et al., 2013; Bikard et al., 2014; Gantz et al., 2015; Hisano et al., 2015; Jakočiūnas et al., 2015; Hammond et al., 2016; Singh et al., 2017a,b). Although it has been less explored in yeast species, the CRISPR-Cas9 system has been recently developed in *S. cerevisiae* for targeting endogenous genomic loci for site-specific mutation and allele replacement (DiCarlo et al., 2013). In addition, Jakočiūnas et al. (2015) have used CRISPR-Cas9-based multiplexing for knocking out up to five different genomic loci (*bts1, ypl062W, yjI064w, rox1*, and *erg9*) in a single transformation step with 100% efficiency in *S. cerevisiae*. With this approach, they have increased the production of mevalonate.

Recent reports on metabolic engineering are based on the manipulation of biosynthetic pathway to redirect the target flux of carbon compounds for the production of industrially important metabolites. A study investigates the deletion of *SIP1* gene of Snf1 kinase complex leading to increase the expression levels of galactose transporter by two-folds to three-folds under glucose-limiting conditions (Shymansky et al., 2017). Recently, Suástegui et al. (2017) have used a multilevel approach for the engineering of an upstream module of aromatic amino acid synthesis pathway and maximized the carbon flux for increasing the production of shikimic acid. In addition, D’Espaux et al. (2017) have produced 1.2 g/L of fatty alcohols that have wide range of applications in manufacturing of detergents, lubricants, surfactants, medicines, personal care products, and potent biofuels by eleven genetic modifications in a parent BY4741 strain of *S. cerevisiae*. In another study, the promiscuous microbial enzyme glycosyltransferase from *S. cerevisiae* was engineered to produce an anticancer molecule (Ginsenoside Rh2), which is naturally produced in *Panax ginseng* and is well-known herbal medicines (Zhuang et al., 2017).

Although many authors have contributed to a great extent in the context of methods and protocols for yeast metabolic engineering, the book “Yeast Metabolic Engineering: Methods and Protocols” edited by Mapelli (2014) has not only discussed the methods, tools, assays, and protocols, but has also encapsulated in-depth the screening and analysis of engineered yeast and its patenting. Eminent scientists in the field of metabolic engineering have contributed in the writing of this book in an easy to follow language, giving it a high level of detail that remains accessible for the reader. It is a timely compilation of the recent advances as well as detailed scope of future possibilities. Moreover, the contributions to this book are based on well-known authors from across the world including Europe, Asia, Australia, and America. The book is divided into four parts (I, II, III, and IV) that contain a total of 18 chapters that cover the molecular toolbox for the manipulation of yeast, assays, design and construction of pathways, analysis of metabolites by analytical methods, genome-scale modeling, and patenting, as well as regulatory issues.

The Part I of this book emphasizes the tools that have been used for the genetic manipulation of yeast strains. It begins with a brief introduction of several categories of selection markers that include prototrophic markers (*ADE1, HIS2, LEU2, AURA3*), carbon or nitrogen source-based selection markers (*amdS, FCA1, GAP1, LSD1*), autoselection system (URA3 system, *CDC4, CDC9, MOB1*), drug resistance markers (*CUP1, SFA1, ble, kan*), and counter-selectable markers [URA3 system, *PKA3* (*TPK2*), *amdS*]. These markers have been employed for the genetic manipulation of yeast strains for basic and applied research. It covers tools for modification of gene expression at transcriptional level to change the metabolic flux of compounds and improve coordination among multiple pathway for producing important metabolite. The authors have focused on natural and synthetic promoters of *S. cerevisiae* that contain the basic structures of yeast core endogenous promoters and upstream *cis*-acting promoter elements and include recent developments in the engineering of promoters with tailor-made properties. The authors have pointed out the challenges in promoter engineering before the formulation of tailor-made properties and describe rational as well as random methods for construction. How these methods can be employed for the modification of endogenous and hybrid promoters to customize promoter characteristics. It also describes how the transcriptional control is of great advantage for metabolic engineering and highlights some limitations that cannot be overcome by promoter selection or engineering.

In addition, the authors have described an alternative yeast strain, *Hansenula polymorpha*, which is a more attractive chassis for metabolic engineering and heterologous recombinant protein production than the baker’s yeast *S. cerevisiae*. It also mentions three strains of *H. polymorpha* (CBS4732, NCYC495, and DL1) commonly used for basic and applied research. The book is also providing practical information about the preparation of media and solutions for DNA isolation, cloning, and transformation, and choice of selective media based on carbon and nitrogen sources. It refers to electroporation as the efficient method for transformation in *H. polymorpha* and describes the gateway technology for the construction of expression vectors for both gene deletion and interaction study. There are other yeast strains of interest, such as *Zygosaccharomyces bailii* which is currently used for industrial applications due to its high stress tolerance properties. In this light, there is a detailed discussion in regard to the acid-tolerance property of *Z. bailii* and its auxotrophic ability that reduces the dependency upon common selection markers. This book provides with protocols for this particular strain and justifies the importance of further development of molecular tools and protocol optimization.

Currently, there are a wide range of synthetic biology tools such as promoters, RBS, transcription terminators, and plasmids available for the genetic manipulation of *Z. bailii*. This strain can be a better choice for the over-production of recombinant proteins because it outperforms the capability of *S. cerevisiae* for the secretion of proteins. Therefore, *Z. bailii* has potential as an established microbial cell factory. Furthermore, the authors have stated electroporation as method with the highest efficiency of transformation, followed by the LiAc-ssDNA (lithium acetate) transformation protocol for liquid cultures together with transformation protocols for plated cultures. The book reviews yeast, *Pichia pastoris* that is also presented as an attractive strain for metabolic engineering. It is more metabolically active and provides a benefit with respect to marker genes due to its auxotrophic nature. The availability of genetic tools for the modification of *P. pastoris* demonstrates its value as an alternative to *E. coli* and mammalian cell lines for the production of recombinant proteins. In this regard, the book covers almost all existing “*Pichia* tools” with a particular focus on different strains ranging from expression strains, methanol-utilizing strains, protease-deficient strains, and glycoengineered ones. Different promoters and signal sequences are also described. The authors explain briefly about the newly generated strains GS115 (*his4*), GS190 (*arg*), JC220 (*ade1*), JC254 (*ura3*) from wild-type strains CBS7435 (NRRLY-11430), DSMZ 70382 (CBS 704), and X-33. Methanol is an important carbon and energy source for *P. pastoris* whose utilization depends upon its distinct phenotypes including *Mut*^+^ (*AOX1 AOX2), Mut^S^* (*aox1 AOX2*), and *Mut*^−^ (*aox1 aox2*). A strain characteristic for its protease deficency such as SMD1168 provides a way to tackle the problem of proteolytic product degradation. In regard to vectors, the book is also dedicated to the description of new vectors (including PICZ, pGAPZ, and pPIC3.5K). Those vectors have the capability to produce multicopy clones with an increased level of gene expression that can be employed for gene knockout experiments. This part of the book explains the crucial role of gene expression, promoters, and secretion signals. There is no genome-editing tool that has been mentioned here, and this is a missing point in this book.

In metabolic engineering, it is important to screen for the best performing strains and this is not possible if the expression of target genes remains unknown. This part focuses on screening protocols that are employed to identify the clone that can express a protein of interest. Direct evolution techniques (Cobb et al., 2013) allow us to create high tolerance strains that can be directly used in metabolic engineering for industrial applications in order to improve the productivity of targeted molecules. The identification and screening of metabolites are challenging issues that can be resolved in the near future by designing of riboswitches or calorimetric assays to identify the top producer strains. This part also elucidates the methods for screening of enzyme libraries in *S. cerevisiae* and details of how fluorescent-based synthetic RNA switches can be employed for screening engineered enzymes. It also provides the protocols for fluorescence measurements, including highly sensitive plate-based measurements using flow cytometry and the less sensitive FACS. Apart from this, it discusses a crucial aspect of protein engineering with numerous applications, ranging from the high throughput screening of protein libraries to the construction of whole cell biocatalysts. A novel system of cell surface display represents a great prospect as a molecular tool, and methods of harnessing yeast cells with cell surface peptides are described. It also mentions the feature of analyzing surface proteins by allowing the cells to remain intact, which makes the system of paramount interest. The protein α-agglutinin is referred as an example of an ideal cell surface display protein target in yeast.

This part not only describes the tools and protocols to employ in these systems but also the scale-up process that moves from laboratory-generated variants toward the industrial-scale production. Even though yeast has the potential to efficiently integrate foreign DNA into its genome and modification of laboratory strains is trouble-free, the application of those strains in the industrial environment remains incompatible. The development of engineered strains in the context of a specific industry is a primary requirement. It also describes some of the marker genes commonly used for development of stable transformants and strategies to accomplish the multiple modifications in yeast. A technique named “*delitto perfetto*” (Storici and Resnick, 2006) utilized for site-directed mutagenesis in yeast has been described in detail.

There is also an explanation of evolutionary engineering as a principle that develops industrially important strains by inverse metabolic engineering. The evolutionary engineering approach evolves strains with resistance to environmental and metabolic stress, plasmid stability, and other general physiological properties, such as competitive fitness, and robustness at the industrial scale. Thus, it is perceived as advantageous over conventional metabolic engineering. In addition, experiments for the selection of modified strains *via* batch or chemostat cultivation are described. Finally, Part I provides a method to determine a strain-specific parameter in *P. pastoris* that can affect the dynamics of batch cultivation systems. The feeding profile is set on the basis of a specific character of the strain to obtain maximum productivity.

The second part of this book (Part II) covers the analytical tools and techniques that are used to explore and determine metabolic features in yeast. It offers a descriptive protocol for metabolome analysis of yeast culture by the use of GC–MS (gas chromatography–mass spectroscopy). It also provides a simple and robust method to harvest and quench yeast cultures and to extract intracellular metabolites by a freeze–thaw method (Smart et al., 2010). The book gives a special emphasis on metabolic flux analysis to quantify the intracellular biochemical reaction rates by tracing stable isotopes, particularly ^13^C (Wiechert, 2001), with a detailed protocol from growing yeast on a ^13^C-labeled substrate to NMR (Nuclear magnetic resonance) detection of ^13^C patterns in protein-bound amino acids, with calculation of metabolic fluxes using software, such as MATLAB and 13CFlux2 (Weitzel et al., 2013). Apart from that, this part provides two methods as an illustration for metabolic flux analysis in *P. pastoris*, namely a Meta-Analysis Package for R (METAFoR) (for growing yeast on glucose and glucose–methanol) and the global iterative fitting methods (for growing yeast on glycerol–methanol) are discussed. In addition to that it is pointed out that pathway activity profiling (PAPi) (Aggio et al., 2010) which is a robust tool that can use the most popular Kyoto Encyclopedia of Genes and Genomes database and the metabolomics dataset for estimating and comparing metabolic pathways between different environmental conditions by using R-software. A simple step-by-step protocol for the PAPi tool offers a better analysis of metabolic pathways. This part ends with the use of the whole genome sequence of the yeast to perform quantitative trait locus mapping (Deutschbauer and Davis, 2005) through applying appropriate protocols.

Metabolic engineering is a powerful approach to be employed if the aim is to generate desirable products, and genome-scale metabolic models prove to be the best choice. In Part III the authors describe some of the widely used genome-scale metabolic engineering models such as iMM904, SpoMBEL1693, iNL895, iSS884, iSS884 for the yeasts *S. cerevisiae, Schizosaccharomyces pombe, P. pastoris*, and *Pichia stipitis*, respectively, that can be used as templates for construction of new genome-scale models of yeast by using the RAVEN (Reconstruction, Analysis, and Visualization of Metabolic Networks) toolbox (Agren et al., 2013). It also explains COnstraint-Based Reconstruction and Analysis (COBRA) (Schellenberger et al., 2011), a computational toolbox for easy model construction that includes some algorithms such as OptStrain and BNICE for the improvement of metabolic engineering. It is also shown how the production of desired products could be increased by re-routing of intracellular fluxes or by identifying the gene deletion targets using genome-scale metabolic engineering models. They provide a quick overview of methods that have been used in the construction of genome-scale metabolic functional models. An example of yeast *S. cerevisiae* used for vanillin production is delivered as a case study. Finally, Part IV describes patenting, ethical, and regulatory issues. It briefly describes patents and the significance for the motivation and protection of researcher’s long-haul endeavor for their research work and intellectual property. Some of the patents and innovations in the area of yeast biology have been described.

We expect that the future version of this book should contain more novel biosynthetic pathways in yeast for the production of chemicals, fuels, therapeutics, and more. We should also expect discussion of genome-editing tools and their potential applications for the better understanding of yeast biology and their use in synthetic biology and metabolic engineering. In conclusion, this book is an excellent and informative text for metabolic engineering experiments, and the editor has made an impressive compilation of almost all the methods for manipulation of yeast strains and the consequent development of strains of industrial significance with great simplicity. The central idea behind this book is to highlight the aspects of metabolic engineering in a way that can help future investigators, researchers, students, and stakeholders to perform research with greater ease. One of the unique features in this book is its protocols for manipulation of yeast cell. The knowledge provided in this book can be implemented to produce desirable metabolic products. To augment the production of such desirable products, there is a pressing need to further develop or employ well the existing tools and technologies.

Although a lot of successes have been made in the metabolic engineering of yeast, even with all of our information and knowledge much remains in this field to explore and expand. This book focuses on the most recent and technological advancement in yeast metabolic engineering. Therein, it is an excellent basis from which scientific knowledge can grow and widen in the fields of metabolic engineering. By compiling and summarizing the current knowledge on yeast metabolic engineering, the have provided us with a rich literary text of excellent depth, clarity, and coverage. This book will play a key role for the better basic and technological understanding of the yeast and its uses in industry for production of biomolecules and as a toolbox for the acceleration of yeast metabolic engineering.

## Author Contributions

NG, HP, SP, RR-G, and VS have designed and written the manuscript. NG, HP, and SP have equally contributed to this work. VS has supervised and finalized the final version of the manuscript.

## Conflict of Interest Statement

The authors declare that the research was conducted in the absence of any commercial or financial relationships that could be construed as a potential conflict of interest.
